# Effective Removal of Cr(VI) from Wastewater Using Biochar Derived from Walnut Shell

**DOI:** 10.3390/ijerph18189670

**Published:** 2021-09-14

**Authors:** Tanzeela Kokab, Hafiza Sumbal Ashraf, Muhammad Bilal Shakoor, Asim Jilani, Sajid Rashid Ahmad, Muzaffar Majid, Shafaqat Ali, Nazar Farid, Rana A. Alghamdi, Diana A. H. Al-Quwaie, Khalid Rehman Hakeem

**Affiliations:** 1 College of Earth and Environmental Sciences, University of the Punjab, Lahore 54000, Pakistan; tanzeela.kokab2@gmail.com (T.K.); hafizasumbalashraf@gmail.com (H.S.A.); sajidpu@yahoo.com (S.R.A.); muzaffar.majid@yahoo.com (M.M.); 2 Center of Nanotechnology, King Abdul Aziz University, Jeddah 21589, Saudi Arabia; 3 Department of Environmental Sciences and Engineering, Government College University, Faisalabad 38000, Pakistan; 4 Department of Biological Sciences and Technology, China Medical University, Taichung 40402, Taiwan; 5 National Centre for Laser Applications (NCLA), School of Physics, National University of Ireland Galway, H91 TK33 Galway, Ireland; nazar.farid@nuigalway.ie; 6 Department of Chemistry, Science and Arts College, Rabigh Campus, King Abdulaziz University, Jeddah 21589, Saudi Arabia; raalghamdi3@kau.edu.sa; 7 Department of Biological Sciences, Science and Arts College, Rabigh Campus, King Abdulaziz University, Jeddah 21589, Saudi Arabia; dalquwaie@kau.edu.sa; 8 Department of Biological Sciences, Faculty of Science, King Abdulaziz University, Jeddah 21589, Saudi Arabia; kur.hakeem@gmail.com

**Keywords:** biochar, chromium, sorption experiments, walnut shell, XPS

## Abstract

Heavy metals are the major concern of the modern age. Among the heavy metals, chromium (Cr(VI)) is regarded as a highly toxic heavy metal released largely from leather tanning operations. To remove such high concentrations of Cr(VI), an advanced method is required urgently. Thus, biosorption using biochar, which is an organic material produced from various sources such as walnut shell, can be applied successfully for Cr(VI) abatement. The major objectives of this experiment were the remediation of the Cr(VI) heavy metal using walnut shell biochar and checking of the effect of pH, biochar dosage, Cr level, and shaking time. Remediation of Cr(VI) using walnut shell biochar was proved to be effective and removed the maximum concentration of Cr(VI) up to 93% at pH 5.5, 2 h agitation time, and the biochar amount of 1.1 g L^−1^ from an aqueous solution. Equilibrium modeling demonstrated that the chemisorption process was involved in adsorption of Cr(VI). The surface of the biochar was porous and provided numerous sites for Cr(VI) attachment, which was also confirmed by the presence of Cr(VI) onto the biochar after adsorption. Hence, the use of walnut shell biochar was highly effective as a sorbent, which could conveniently be applied to small-scale as well as large-scale levels.

## 1. Introduction

Metals having a density higher than 4 g/cm^3^ or five times higher than water are known as heavy metals, i.e., copper, chromium, manganese, lead, mercury, zinc, iron cobalt, cadmium, arsenic, nickel, silver, and platinum. Heavy metals are abundantly present in soil and aquatic ecosystems, and small portions are present in the atmosphere as particles or vapors [1]. In the environment, heavy metal pollution is a modern concern as it is continuously contaminating water and soil, and adversely affecting living creatures of the biosphere. Heavy metals are non-biodegradable and their bioaccumulation is increasing gradually in the food chain, thus deteriorating quality of living.

The minor to major contamination of the environment due to chromium (Cr) is the result of human-induced activities. The industrial revolution started due to human advancement and after this revolution, chromium began to increase in the atmosphere, environment, soil, and water. The revolutionary industries that require Cr include the leather tannery, steel industry, electroplating industry, lime kilns, cement industry, and metallurgy. These artificial sources are naturally increasing the chromium levels in environments to toxic levels [2].

In the earth crust, the concentration of Cr is abundant among all the elements. The normal level of chromium is 125 mg g^−1^. In the periodic table, it is found at the 24th position in group 6B of the transition metals. In the rocks, it is at the 24th number according to the concentration present in rocks. The amount of Cr in sea water ranges from 5 to 8 µg L^−1^; in soil it ranges from 1 to 300 mg g^−1^; and in lakes and rivers its value lies between 26 µg L^−1^ and 5.2 mg L^−1^ [3]. Due to the higher solubility feature of chromium (Cr(VI)), it causes more toxic effects in the environment (human and animals) than chromium (III) and has been categorized as a probable carcinogen and mutagen [4].

Chromium (III), which is a common and stable form, persists as chromite ore, while Cr(VI) is a dynamic and highly soluble form of Cr [5]. Chromium (VI) is highly unstable, reactive, and oxidizes easily [6].

There are many exposure pathways for chromium exposure including (a) ingestion, (b) inhalation, (c) direct contact in industry, and (d) orally. The major dose of chromium is found in meat, fish, vegetables, and fruits, which are flourished with chromium-containing water [7]. Humans can have or experience skin cancer, ulcers, gastrointestinal disorders, genetic mutations, effects on the immune system, respiratory diseases (bronchitis), and liver and kidney disorders after exposure to Cr(VI). Plants can experience reluctant growth and aquatic life would experience respiratory issues. Fish can face reduced activity of gills [2]. The EPA has set a limit for Cr(VI) at 0.01 mg/L and 0.05 mg/L for drinking water and domestic water, respectively [8].

To minimize the risks of Cr(VI) contamination, the only way to solve this problem is remediation. The Cr(VI) is reduced into chromium (III), as it is a less toxic form. Conventional technologies help in the removal of this inorganic effluent (chromium) but these are all major causes of secondary pollutants and the generation of toxic sludge [2].

In the past few years, many conventional techniques have been used for the abatement of Cr(VI), for example, including chemical precipitation, flotation, ion exchange, reverse osmosis, solvent extraction, oxidation and ultrafiltration, and the membrane hybrid process. High energy, capital expenses, and chemical requirements are the key disadvantages of these techniques [9]. In contrast, in biosorption, the biomaterials are antioxidant and are benign substitutes to treat hexavalent chromium. They have a low cost and are both efficient and environmental-friendly [10]. Biochar is a charcoal-like substance that is made by burning organic materials in the absence of oxygen [11].

There are so many other materials, such as rice husk, fruit peels, wheat straw, bamboo husk, coconut husk, peelings of jack fruit and egg shells, etc., which can be used to prepare biochar [12]. Biochar is preferred in comparison to other conventional techniques due to its environmental benefits. The decreasing cost, less water pollution, little/no organic waste, and agricultural-friendly nature are the major advantages of biochar. It also secures food by increasing crop yield and retaining water [13].

Not only because of its less cost, biochar has other effective properties that make its use favorable. Biochar is a carbon (C)-rich material. It has many functional groups such as hydroxyl (OH^−^), carboxylic (COOH^−^), alkyl, and the phenyl and amino functional group. These functional groups are negatively charged, which assists in the Cr absorption by replacing the ions [14]. The major properties are the surface area and porosity of biochar. When biochar is prepared in the absence of oxygen, dehydration leaves many pores on the surface of biochar. These pores help in the adsorption of heavy metals such as Cr(VI) [15]. Walnut production is estimated to be at 3.46 million tons per ~1 million ha globally, with a mean value of 3.1 tons ha^−1^. In Pakistan, the walnut is grown at about 3.12 thousand ha, yielding 21.6 thousand tons with a mean production of 6.9 tons ha^−1^. Consequently, a huge amount of walnut shell is also produced in Pakistan as well as all over the world [16]. Thus, to make better use of this low-cost and readily available solid organic waste, it is suggested to employ for biochar production. Moreover, to our knowledge, little/no data is available on the use of walnut shell biochar for Cr(VI) abatement in water. Therefore, the current study was planned (a) to examine the removal of Cr(VI) from wastewater using walnut shell biochar; (b) to assess the effect of various parameters, including pH, chromium concentration, biochar dose, and desorption; and (c) to identify the adsorption mechanism using equilibrium modeling as well as surface morphology and chemical composition by SEM-EDS and XPS.

## 2. Materials and Methods

### 2.1. Chemicals and Standards

The batch sorption experiment was carried out by employing standard chemicals. The chromium standard solution was made by using desired amount of potassium dichromate in the distilled water. Nitric acid solution was prepared in order to wash the glass wares and plastic wares after their washing with distilled water 3 times. All the analyses were carried out with the help of an atomic absorption spectrophotometer.

### 2.2. Biomaterial Collection and Biochar Preparation

For the preparation of biochar, walnut shell (WS) was obtained from the local market of Lahore, Pakistan. Firstly, it was necessary to remove the moisture content from the WS. For this purpose, the WS was subjected to direct sunlight for 2 to 3 days. As the WS became hard, it was passed through a shredder to cut it into smaller pieces. In the next step, the WS was oven-dried at 65 °C for 72 h. When the moisture content dried totally, the walnut shell biochar (WSB) was prepared by moving the material into the muffle furnace at 450 °C for 2 h, followed by subsequent cooling. Once the temperature became normal, it was converted into powder form, which was then sieved by using sieve of 2 micrometers in size [17].

### 2.3. Surface Characterization

The surface functional groups were determined by employing FTIR spectroscopy (Nicolet iS10, Thermo Fisher Scientific, Waltham, MA, USA). The morphology and elemental composition were determined by a field emission scanning electron microscope (FESEM- JEOL- JSM7600-F, Japan) with an energy dispersive X-ray (EDX-Oxford). The sample (WSB) was mounted over the copper stub with carbon tape while the working distance was kept at 8 mm. The surface composition of Cr(VI) with loaded and unloaded WSB was obtained through X-ray photoelectron spectroscopy (XPS) (ESCALAB 250-@Al-Ka-1486.6 eV, Thermo Fisher Scientific, USA). Moreover, the sample (WSB) was kept under vacuum (10^−6^) overnight to minimize the chance of surface contamination. The working X-ray spot size of 200 μ was used during the survey scan.

### 2.4. Batch Sorption

Falcon bottles of 50 mL were used to carry out the batch sorption procedure. For the electrolytes supply, 0.01 molar solution of NaCl was used. The experimental work was done at an optimum temperature at various environmental conditions using the desired initial Cr(VI) level (5–200 mg L^−1^) as described below.

### 2.5. pH

For the pH study, it was necessary to maintain the pH of the NaCl solution that was used as the electrolytic solution. Thus, pH was maintained by adding NaOH sodium hydroxide and HCl (hydrochloric acid). In addition, 110 mg L^−1^ of Cr and 1.1 g L^−1^ of the WSB dose in a 25-mL NaCl solution were added. The pH value was set from 3 to 8 in six Falcon bottles. Then, the bottles were subjected to shaking in an orbital shaker for 2 h. Samples were carefully filtered and the Cr in samples was analyzed.

### 2.6. Isotherm Study

The initial Cr concentration values (5, 10, 30, 50, 70, 90, 100, 110, 150, 170, and 200 mg L^−1^) were added into the NaCl solution. The amount of the biochar dose was 1.1 g L^−1^ in all the samples, with a pH of 5.5. After that, the samples were subjected to shaking for 2 h in an orbital shaker. Then, they were filtered and the remaining extract was subjected to Cr detection.

### 2.7. Biosorbent Dose

The biosorbent dose was studied to test the effect of different doses of biochar. Different doses (0.1, 0.2, 0.3, 0.5, 0.7, 0.9, 1, 1.1, 1.2, and 1.3 g L^−1^) were mixed with 110 mg L^−1^ Cr concentration in all the samples at pH 5.5. The samples were subjected to shaking for 2 h in an orbital shaker. Then, they were filtered and the remaining extract was subjected to Cr analyses.

### 2.8. Contact Time (Kinetic Study)

The contact time was changed while maintaining the constant concentration of the biosorbent dose (1.1 g L^−1^) and Cr (110 mg L^−1^), and the contact time for shaking varied during 1–240 min. After centrifugation, samples were filtered with filter paper and the Cr in samples was analyzed.

Mathematical calculations for Cr sorption onto WSB

Mathematically, the percentage of Cr is determined as expressed in (Equation (1));

(1) $$\%age\;removal=\frac{C_{o}-C_{e}}{C_{o}}\times 100\;$$

where the

initial concentration (Cr) = *C_o_* (mg L^−1^) and

equilibrium concentration Cr = *C_e_* (mg L^−1^).

Cr adsorbed by the mass of the biochar = (*q_e_*) is calculated by (Equation (2)):

(2) $$q_{e}=\frac{\left(C_{o}-C_{e}\right)V}{m}\;$$

where

*q_e_* = the equilibrium sorption potential in mg g^−1^;

*V* = the solution volume; and

*m* = the biochar weight.

### 2.9. Equilibrium Modeling

The chromium sorption mechanism in the WSB was examined by applying modeling techniques such as kinetics and isotherm (Supplementary Material).

## 3. Results and Discussion

### 3.1. pH

**Figure 1:**
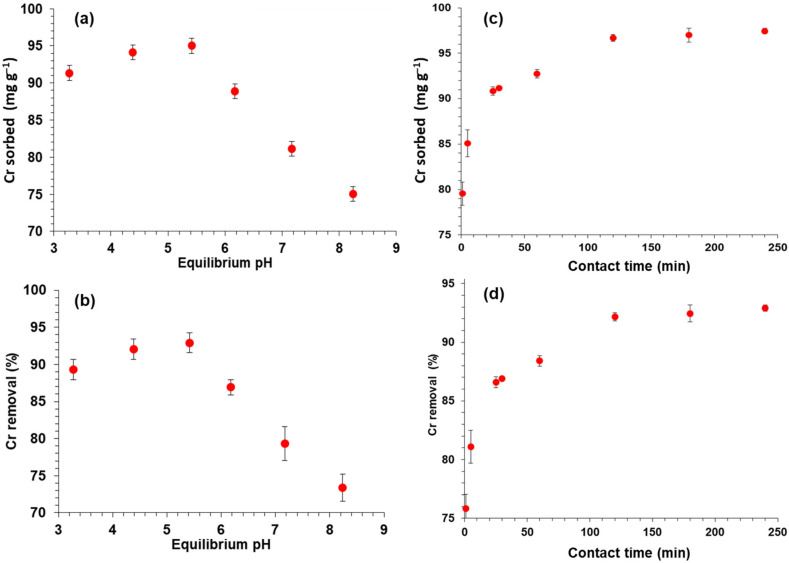
Adsorption of chromium on WSB: (**a**,**b**) pH effect and (**c**,**d**) contact time effect.

The parameter of pH of the solution has significant importance during the bio-sorption process in terms of the biosorption of different metal ions. The variation in pH affects the process of diffusion, charges at the surface, and linking properties of metal and metalloid ions after speciation. The charge on the biosorbent surface can be maintained by changing the pH. As the charge on biosorbent surface modifies, metal and metalloid ions’ specification will also vary. Thus, variation in pH has critical impacts on chromium adsorption and chromium removal. Giving the importance of pH, the sorption was carried out by changing the pH within the range of 3 to 8. This sorption was carried by using WSB (Figure 1a,b). At the lower pH (acidic) of 3.2, 91 mg g^−1^ chromium was adsorbed. As pH increased from 3 to 4, the adsorption of chromium was significant and at pH 4.4, 94 mg g^−1^ chromium was adsorbed; as pH increased up to 5.5, however, a maximum amount of chromium, i.e., almost 94.5 mg g^−1^ (93% Cr removal), was adsorbed.

The pH above 5.5 to 6.2 showed that sorption of chromium decreased gradually from 94.5 to 89 mg g^−1^. As a gradual increase occurred in the pH, the sorption declined. It was at the minimum (74.5 mg g^−1^) at the highest pH of ~8.

This proves that the sorption was highest at an acidic pH and lower at a basic pH. The highest sorption value observed was at pH 5.5. The main reason for this maximum sorption is neutralization. Hydroxyl ions (OH^−^) were neutralized due to an excess amount of hydrogen (H^+^) ions, which encourages the diffusion of dichromate ions and the result is the adsorption of ions on the biochar surface. At an acidic pH, the biochar sorbent surfaces were positively charged, while chromium ions were negative charged, thus pH 5.5 was sufficient to interact with chromium ions. As pH increased above 5.5, however, the hydroxyl ions’ concentration was increased due to excess OH^−^ ions and the surface of the biochar and chromium ions in the solution repelled each other due to repulsive forces [18].

### 3.2. Contact Time

The Figure 1c,d shows that the minimum amount of Cr was adsorbed below 10 min. As the contact time increased up to 30 min, 91 mg g^−1^ of Cr was adsorbed and the rising contact time enhanced the amount of adsorbed Cr. When the time of 1 h was reached, a significant amount of chromium, specifically 96.5 mg g^−1^ (93% Cr removal), was adsorbed, but a gradual increase took place in the sorption. After the contact time of 2 h, a negligible change occurred when time was increased to 4 h. Thus, this proves that the biosorbent solution has a direct relation with contact time and that the capacity of the biosorbent is increased with contact time. As contact time increased, more pores were available for the binding of metal ions. As a result, more Cr(VI) were diffused onto the sites of the biosorbent. Prolonged increased in contact time, however, could not increase the sorption because competition began between the metal ions due to lessening of the surface area fraction on the biochar dose [19].

### 3.3. Iinitial Chromium Concentration

**Figure 2:**
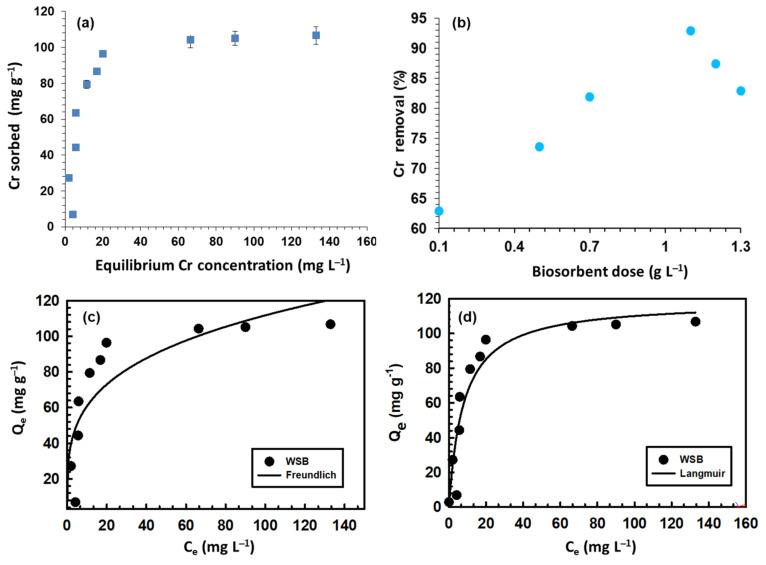
Adsorption of chromium on walnut shell biochar: (**a**) initial sorbate concentration effect; (**b**) biosorbent dose effect; and (**c**,**d**) isotherm modeling of equilibrium data.

The other important parameter that helps in determining the chromium removal is the initial concentration of chromium. Different concentrations of chromium were added at 5.5 pH with a contact time of 2 h (Figure 2a). Chromium concentration values were 5, 10, 30, 50, 70, 90, 100, 110, 150, 170, and 200 mg L^−1^ and there is a direct relation between the chromium concentration and removal efficiency. At a very low concentration of 5 mg L^−1^, a minimum amount was adsorbed. According to the abovementioned concentration values, as the amount increased from 5 to 100 mg L^−1^, the gradual increase in chromium adsorption took place. At the concentration of 110 mg L^−1^, about 104.2 mg g^−1^ Cr adsorption was obtained, but after that the adsorption became almost constant. The reason for this decline could be that large amounts of negatively charged chromium ions did not interact with the surface of the biosorbent due to disturbances in the binding forces [20]. Another reason could be the fixed amount of the biochar dose, although the increasing concentration of Cr resulted in a relatively reduced adsorption capacity of WSB.

Moreover, the decreasing adsorption with the rising initial Cr(VI) concentration might be due to the fact that the biochar possessed a finite amount of active adsorption cavities and these were almost saturated at a definite Cr(VI) level; thus saturated biochar might not adsorb additional Cr(VI) ions from water [18].

### 3.4. Effect of the Biochar Dose

In order to study the removal efficiencies of chromium by WSB, this parameter is important because it supports the intake of metalloids from the solution. In the contact time and pH parameter, the biosorbent dose was kept constant, thus varying doses gave different results of sorption. Figure 2b shows that chromium removal was lowest at a very low dose. As the biosorbent dosage enhanced from 0.1 g L^−1^ to an optimum value of 0.5 g L^−1^, nearly 74% chromium was removed and it further increased up to 81% at the biochar dosage of 0.7 g L^−1^ due to the availability of more exchangeable sites [21]. The gradual increase in the removal efficiency took place at the dose of 1.1 g L^−1^. Almost 93% of chromium was removed at this dose. The reason behind this might be the fact that the enlarged surface area was present for the removal of maximally negatively charged chromium ions. The further increase in the dose up to 1.2 g L^−1^ and 1.3 g L^−1^ did not increase the adsorption of chromium ions. This could be due to the fact that the further increase in amount can increase the surface area but the amount of chromium in the solution will not change, thus the amount of chromium, which has to be adsorbed in the biosorbent dose, was already adsorbed. In addition, the particles of the biosorbent dose at the sorption sites overlapped and overcrowded, causing a reduction in the chromium adsorption [22]. Moreover, the decline in adsorption with the enhancing biochar dose could be due to unsaturation of the biochar adsorption active sites, as well as due to an increase in the diffusion pathway occurring because of aggregation of the biochar particles [23].

### 3.5. Isotherm Modeling

The isotherm equations were fitted to Freundlich and Langmuir models as illustrated in Figure 2c,d and the model parameter values are given in Table 1. It was observed that the Freundlich model reported the *n* value during the Cr(VI) adsorption on WSB as <1, suggesting that adsorption was a favorable procedure (Table 1). However, the *R*^2^ value obtained in the Langmuir model was higher (0.89) compared to *R*^2^ calculated in the Freundlich model (0.78) (Figure 2c,d). The outcomes of the isotherm modeling suggested that Cr(VI) adsorption was due to monolayer adsorption, which was controlled by the chemisorption process.

### 3.6. Kinetic Modeling

**Figure 3:**
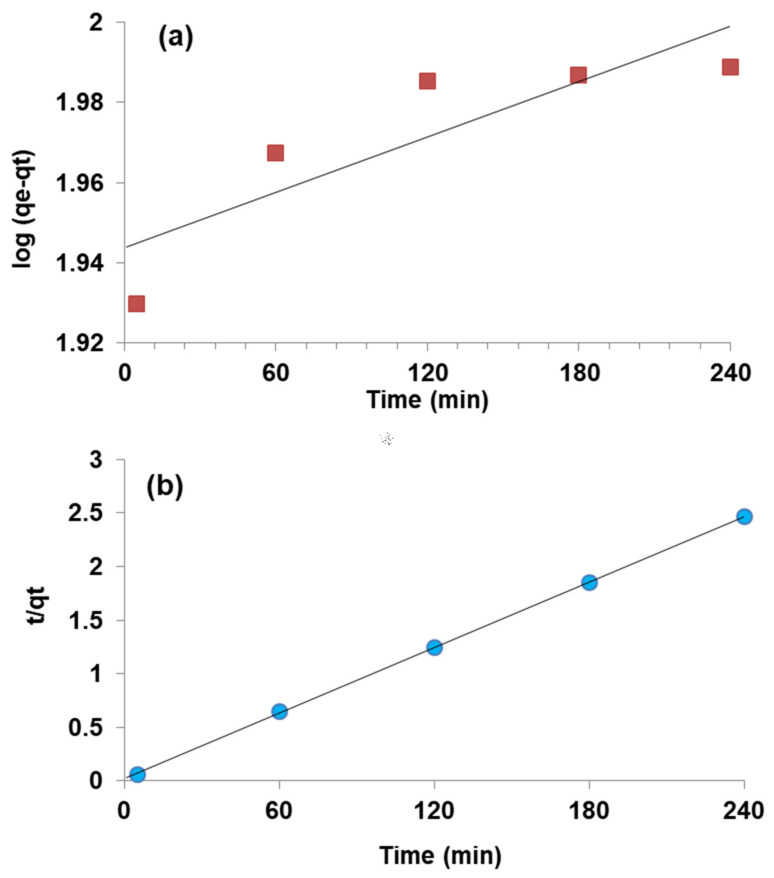
Kinetic modeling graphs of chromium adsorption on the walnut shell biochar: (**a**) PFO (**b**) PSO.

Adsorption on the WSB was explained through kinetic models including the pseudo first-order (PFO) and pseudo second-order (PSO) to unravel the adsorption rate on the WSB (Figure 3a,b). Table 2 demonstrates the values of Cr(VI) adsorption on the WSB obtained in the modeling. The comparison of *R*^2^ values of PFO and PSO showed that the PSO model provided the best fit (*R*^2^ = 0.99) compared to the PSO model for Cr(VI) adsorption on the WSB (Table 2). Moreover, the *q_e_* value calculated in the PSO model (100 mg g^−1^) was found to be greater than the PFO model (87 mg g^−1^). It was deduced that the amount of adsorption cavities on the WSB could affect the rate of Cr(VI) adsorption and the chemisorption mechanism was responsible for adsorption, as demonstrated by the best fitting of the PSO model [24].

### 3.7. FTIR

**Figure 4:**
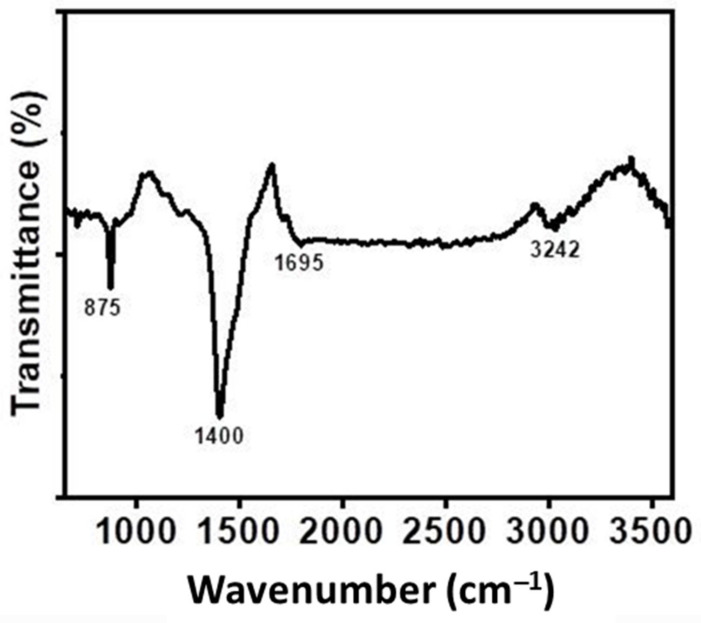
Identification of surface functional groups by FTIR spectra of the walnut shell biochar.

The FTIR spectra of WSB showed that at approximately 3242 cm^−1^ (Figure 4), the characteristic peak for the stretching vibrations of the hydroxyl group (−OH) was prominent [25]. The peak found at 1695 cm^−1^ was denoted as belonging to the group C=O in the carboxylic acid present in hemicelluloses. At 1400 cm^−1^, the peak was assigned to C=O in the carboxylates, quinones, and ketones [26]. The peak at 875 cm^−1^ could be due to C–H vibrations in aromatic compounds. Overall the FTIR data suggests the presence of potential functional groups, which could be helpful for adsorption of Cr(VI) from water.

### 3.8. SEM-EDX

**Figure 5:**
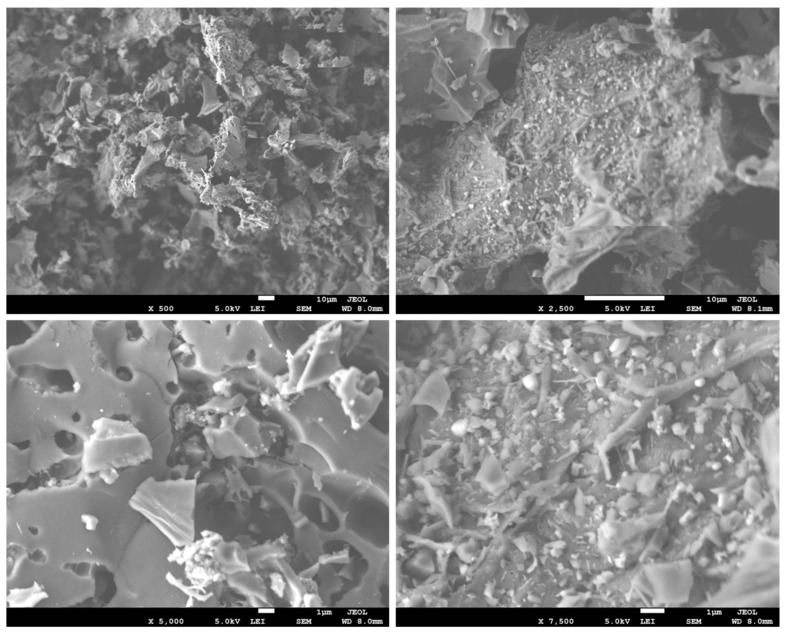
SEM micrographs of the walnut shell biochar.

The surface morphology and porous structure of WSB were explored using SEM analysis. It was noted in Figure 5 that the surface structure of pristine WSB was observed to be rough, irregular, and uneven, possessing numerous macro and micro-pores of different shapes and sizes that provided sufficient adsorption cavities and spaces. Furthermore, the biochar’s rough surface might result in the improved surface area, thereby providing beneficial effects for the adsorption of pollutants [27].

**Figure 6:**
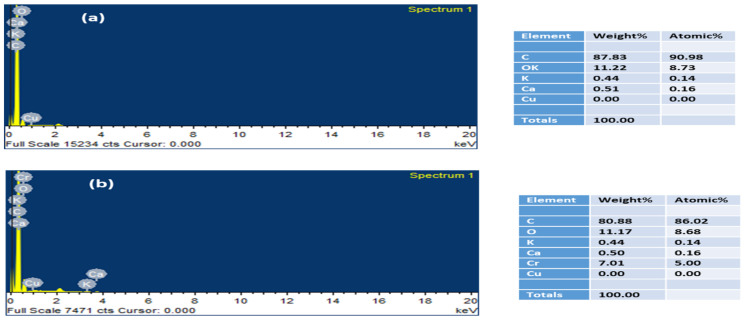
EDX data of chromium adsorption on the walnut shell biochar: (**a**) unloaded walnut shell and (**b**) chromium-loaded walnut shell.

The EDX spectra of WSB before and after Cr(VI) adsorption was obtained, as depicted in Figure 6a,b. For raw WSB, the weight percentages of C, O, and Cr(VI) were 87.83%, 11.22%, and 0%, respectively. When Cr(VI) was adsorbed, these percentages were observed to be 80.88, 11.17, and 7.01 for C, O, and Cr(VI), respectively. The detection of Cr(VI) on the WSB showed that WSB is a potential material for Cr(VI) removal. Moreover, after Cr(VI) adsorption, the O/C ratio enhanced from 0.12 to 0.14, indicating an increment in oxygen-rich surface functional groups, which could be due to Cr(VI) reduction [28].

### 3.9. XPS

**Figure 7:**
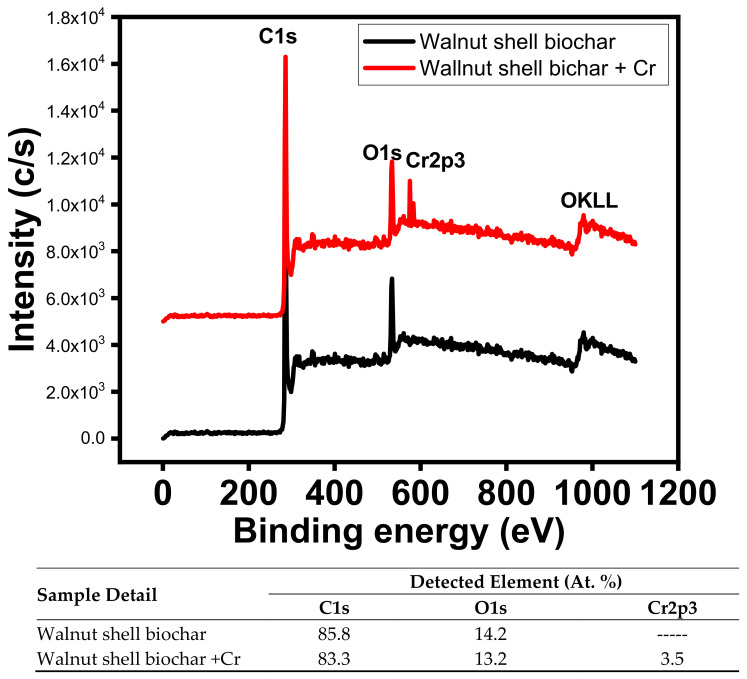
XPS survey scan of chromium adsorption on the walnut shell biochar.

Figure 7 displays the XPS survey scans of WSB with and without Cr. Distinct peaks of C (1s), O (1s), and Cr (2p3) were noted and Cr was also detected after the adsorption onto WSB. The data reported that the percentage of C and O was 85.8% and 14.2%, respectively, on the surface of WSB.

The adsorption of Cr(VI) resulted in the loading of Cr onto WSB and the percentage of C, O, and Cr was observed to be 85.3%, 13.2%, and 3.5%, respectively. Thus, the presence of a potential percentage of Cr(VI) on WSB confirms the suitability of WSB for the treatment of chromium-containing wastewater.

Table 3 shows the Cr(VI) adsorption efficiencies of biochars used previously. It can be observed that walnut shell biochar (94.5 mg g^−1^) provided better Cr(VI) adsorption than many other biochars used before, such as oak bark biochar (4.6 mg g^−1^), pineapple peel biochar (7.44 mg g^−1^), and water melon peel biochar (69 mg g^−1^).

## 4. Conclusions

The use of walnut shell biochar showed a maximum removal efficiency of 93% for Cr(VI). The maximum removal was attained at 110 mg L^−1^ Cr, 1.1 g L^−1^ WSB amount, and with pH 5.5 and 2 h contact time. Isotherm modeling and kinetic modeling parameters illustrated that the chemisorption was a favorable process for Cr(VI) adsorption. The SEM data showed that WSB possessed a rough and uneven surface with many pore spaces, while EDX analyses successfully detected Cr(VI) on WSB. The XPS results also confirmed the presence of O-rich surface functional groups and efficient Cr(VI) adsorption onto WSB. Thus, WSB is a suitable and economically viable material as walnuts are available easily in the market. It can be employed in future treatment technologies for treating wastewater according to the required environment. In regard to climate change, the major benefit of biochar usage concerns the absence of sludge generation, with no toxic chemicals present as the material is totally organic in nature. That is why it is preferred over all conventional treatment technologies, which are highly costly, produce toxic sludge, and cause disposal issues in the environment.

## Figures and Tables

**Table 1:** Isotherm model values for chromium adsorption on the walnut shell biochar.

Isotherm Models	Parameters	
Langmuir	*Q_L_*	15.02 (mg g^−1^)
	*R* ^2^	0.89
	*K_L_*	0.12 (L g^−1^)
Freundlich	*Q_F_*	32.98 (mg^1−n^ g^−1^ L^n^)
	*R* ^2^	0.78
	*n*	0.26

**Table 2:** Kinetic model values for the walnut shell biochar.

PFO			PSO		
*q_e_* (mg g^−1^)	*k*_1_ (min^−1^)	*R* ^2^	*q_e_* (mg g^−1^)	*k*_2_ (g mg^−1^ min^−1^)	*R* ^2^
87	0.00002	0.75	100	0.01	0.99

**Table 3:** Adsorption capacity of WSB and comparison with other biochars.

Biochar	Cr(VI) Sorption (mg g^−1^)	
Dried sewage sludge	3	[29]
Oak bark	4.6	[19]
Oak wood	3	
Flax shive	14.3	[30]
Pineapple peel	7.44	[31]
*Onopordom Heteracanthom*	37.28	[23]
Water melon peel	69	[32]
Walnut shell	94.5	This study

## Data Availability

Not applicable.

## Supplementary Materials

The following are available online at https://www.mdpi.com/article/10.3390/ijerph18189670/s1, File S1: Equilibrium modeling.
