# Supplementary material for: Effective Removal of Cr(VI) from Wastewater Using Biochar Derived from Walnut Shell
Source: Int J Environ Res Public Health. 2021 Sep 14;18(18):9670. doi: 10.3390/ijerph18189670 (PMC8467037; doi:10.3390/ijerph18189670)
Supplement: Supplementary file 1 [file ijerph-18-09670-s001.zip › ijerph-1336000-supplementary/ijerph-1336000-File S1.pdf]

Kinetic modeling of sorption dynamics

On biosorbents mass, the sorption rate of Cr(VI) was examined by applying pseudo-first order and pseudo-second order. Following equations 1 and 2 were used for pseudo-first order which is grounded on the capacity of solid biosorbent.

$$\log(q_e - q_t) = \frac{\log q_e - k_1 t}{2.303} \quad (1)$$

In the above equation Cr (VI) sorbed amount at any time *t* (mg g^{-1}) is mentioned with qt, equation constant rate with unit min^{-1} is mentioned as k_1 and sorbed Cr amount at equilibrium with unit mg g^{-1} is named as q_e. Plotting log ($qe - qt$) vs *t* was used to determine k_1 which is the constant rate of biosorption.

The kinetic model, pseudo-second order was utilized which suggest that sorption rate is proportional to the square of the number of remaining free sorption active sites. Following is the equation (Eq 4) to describe the pseudo-second order.

$$\frac{t}{q_t} = \frac{1}{k_2 q_e^2} + \frac{t}{q_e} \quad (2)$$

In the above equation of pseudo-second order. equilibrium constant rate with unit (g mg^{-1} min^{-1}) is mentioned with k_2. The intercept of $1/(k2qe2)$ and slope of $1/qe$ are linear between the relationship of *t* and *t/qt* [29, 30].

Models of sorption isotherm

The mechanism of Cr sorption on the biosorbent surface was described by applying isotherm equilibrium models of Temkin, Freundlich, Dubinin-Radushkevich and Langmuir which are based on two parameters. Multi-layer sorption or sorption on heterogeneous surfaces was described by Freundilich model with the equation described below (Eq. 3):

$$Q_e = Q_F C_e^n \ (3)$$

In the equation linearity related parameter was mentioned with n and capacity of Cr sorption with units mg^{1-n} g^{-1} L^n was shown with Q_F.

Langmuir model was used to describe the monolayer adsorption and sorption on planar surfaces with the Eq. 4 given below:

$$Q_e = Q_L K_L C_e (1 + K_L C_e)^{-1} \ (4)$$

In the equation mentioned above, K_L is representing the constant of sorption equilibrium with unit (L mg^{-1}) and maximum Cr(VI) sorbed amount (mg g^{-1}) was described by Q_L.
